# Supplementary material for: Metabolically flexible microorganisms rapidly establish glacial foreland ecosystems
Source: Nat Commun. 2025 Nov 26;16:11634. doi: 10.1038/s41467-025-66734-4 (PMC12749492; doi:10.1038/s41467-025-66734-4)
Supplement: Supplementary file 1 — Supplementary Information [file 41467_2025_66734_MOESM1_ESM.pdf]

Metabolically flexible microorganisms rapidly establish glacial foreland ecosystems

Francesco Ricci[1,2 #], Sean K. Bay[1,3 #], Philipp A. Nauer[4], Wei Wen Wong[4], Gaofeng Ni[1], Luis Jimenez[1], Thanavit Jirapanjawat[1], Pok Man Leung[1,2], James A. Bradley[5,6], Vera M. Eate[4], Montgomery Hall[1], Astrid Stubbusch[1], Beatriz Fernández Marin[7], Asunción de los Ríos[8], Perran L.M. Cook[4], Martin H. Schroth[9], Eleonora Chiri[1,10 *], Chris Greening[1,2,10 *]

[1]Department of Microbiology, Biomedicine Discovery Institute, Monash University, Melbourne, VIC, Australia

[2]Securing Antarctica's Environmental Future, Monash University, Melbourne, VIC, Australia

[3]Department Microbiology, Anatomy, Physiology & Pharmacology, La Trobe University, Melbourne, VIC, Australia

[4]Water Studies Centre, School of Chemistry, Monash University, Melbourne, VIC, Australia

[5]Aix Marseille Université, Université de Toulon, CNRS, IRD, MIO, Marseille, France

[6]School of Biological and Behavioural Sciences, Queen Mary University of London, London, UK

[7]Department of Plant Biology and Ecology, University of the Basque Country (UPV/EHU), Barrio Sarriena s/n, 48940 Leioa, Bizkaia, Spain

[8]Museo Nacional de Ciencias Naturales, Consejo Superior de Investigaciones Científicas, Madrid, Spain

[9]Environmental Systems Science, ETH, Zürich, Switzerland

[10]School of Biological Sciences, Monash University, Melbourne, VIC, Australia

# These authors contributed equally to this work

* Correspondence can be addressed to chris.greening@monash.edu or eleonora.chiri@agromini.org

Zeta diversity decline and retention rate

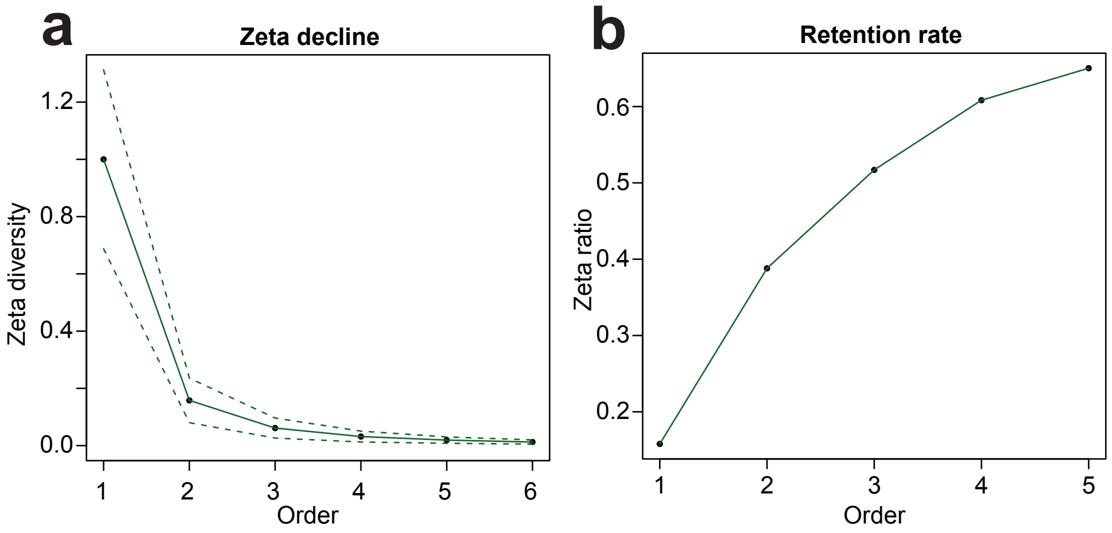

Figure S1 | Multisite community turnover and assembly processes along the Swiss glacial foreland. **(a)** Zeta decline illustrates how the number of shared taxa (ASVs) decreases as more sites are included in the comparison (Zeta Order). **(b)** Zeta diversity ratio showing the rate of taxon retention, highlighting the probability of retaining common taxa over rare ones at any given order as additional sites are included in the comparison (n=15).

Random forest analysis of environmental drivers of selected marker genes

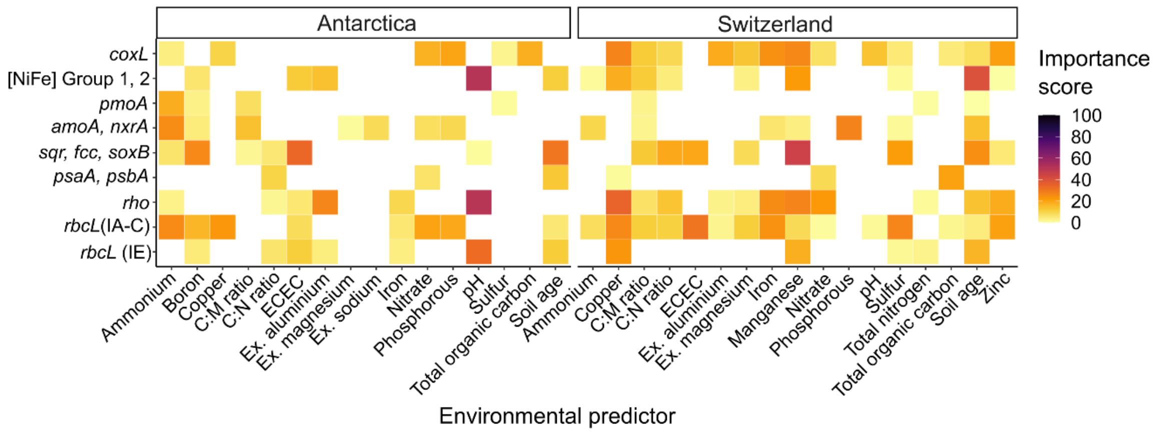

Figure S2 | Random forest analysis showing the influence (importance score) of environmental variables over the relative abundance of metabolic marker genes for trace gas oxidation, nitrification, sulfide and thiosulfide oxidation, phototrophy and carbon fixation recovered from the Antarctic and Swiss glacial forelands.

Biogeochemical assays and power per cell

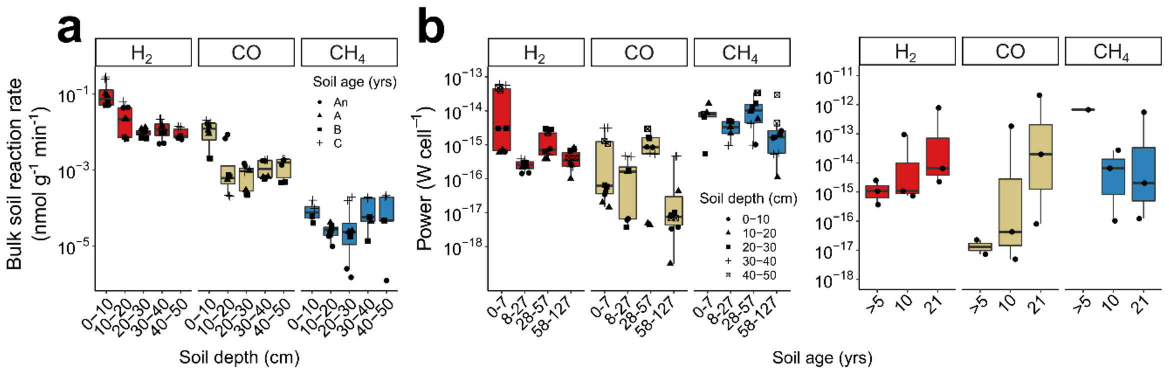

Figure S3 | (a) Bulk soil oxidation rate for each gas, according to soil depth of Swiss samples with shapes showing respective soil age. Measurements were performed with at least four biological replicates per age group. **(b)** Amount of power per cell generated from the oxidation of each trace gas, calculated using thermodynamic models based on ex situ rates measured on five depths for the Swiss glacial foreland and on top Antarctic foreland soil. Measurements were performed with at least two biological replicates per age group.
