# Supplementary material for: Metabolically flexible microorganisms rapidly establish glacial foreland ecosystems
Source: Nat Commun. 2025 Nov 26;16:11634. doi: 10.1038/s41467-025-66734-4 (PMC12749492; doi:10.1038/s41467-025-66734-4)
Supplement: Supplementary file 7 — Reporting Summary [file 41467_2025_66734_MOESM7_ESM.pdf]

nature portfolio

Corresponding author(s): Chris Greening

Last updated by author(s): Aug 3, 2025

Reporting Summary

Nature Portfolio wishes to improve the reproducibility of the work that we publish. This form provides structure for consistency and transparency in reporting. For further information on Nature Portfolio policies, see our Editorial Policies and the Editorial Policy Checklist.

Statistics

For all statistical analyses, confirm that the following items are present in the figure legend, table legend, main text, or Methods section.

n/a	Confirmed	
☐	☒	The exact sample size (*n*) for each experimental group/condition, given as a discrete number and unit of measurement
☐	☒	A statement on whether measurements were taken from distinct samples or whether the same sample was measured repeatedly
☐	☒	The statistical test(s) used AND whether they are one- or two-sided<br>*Only common tests should be described solely by name; describe more complex techniques in the Methods section.*
☐	☒	A description of all covariates tested
☐	☒	A description of any assumptions or corrections, such as tests of normality and adjustment for multiple comparisons
☐	☒	A full description of the statistical parameters including central tendency (e.g. means) or other basic estimates (e.g. regression coefficient) AND variation (e.g. standard deviation) or associated estimates of uncertainty (e.g. confidence intervals)
☒	☐	For null hypothesis testing, the test statistic (e.g. *F*, *t*, *r*) with confidence intervals, effect sizes, degrees of freedom and *P* value noted<br>*Give P values as exact values whenever suitable.*
☒	☐	For Bayesian analysis, information on the choice of priors and Markov chain Monte Carlo settings
☒	☐	For hierarchical and complex designs, identification of the appropriate level for tests and full reporting of outcomes
☐	☒	Estimates of effect sizes (e.g. Cohen's *d*, Pearson's *r*), indicating how they were calculated

Our web collection on statistics for biologists contains articles on many of the points above.

Software and code

Policy information about availability of computer code

Data collection	No commercial, open source or custom code was used in this study to collect data.
Data analysis	No commercial or custom code was used for data analysis. Open source code / software was used as described in the methods and listed here: QIIME 2, R, Phyloseq, iNEXT, VEGAN, zetadiv, DIAMOND, randomForest, Metaphor, CONCOCT, MEGAHit, Vamb, SemiBin2, MetaBat, MetaWrap, CheckM2, dRep, GTDP-Tk, PhyloPhLan, IQ-Tree, ITOL

For manuscripts utilizing custom algorithms or software that are central to the research but not yet described in published literature, software must be made available to editors and reviewers. We strongly encourage code deposition in a community repository (e.g. GitHub). See the Nature Portfolio guidelines for submitting code & software for further information.

Data

Policy information about availability of data

All manuscripts must include a data availability statement. This statement should provide the following information, where applicable:

- Accession codes, unique identifiers, or web links for publicly available datasets
- A description of any restrictions on data availability
- For clinical datasets or third party data, please ensure that the statement adheres to our policy

All sequence data from this study is available at the Sequence Read Archive, with accession numbers PRJNA1178459 for metagenomic sequences, PRJNA1178814 for 16S rRNA gene sequences, PRJNA1206715 for Antarctic MAGs, and PRJNA1206714 for Swiss MAGs. All raw data is provided in the supplementary tables.

Research involving human participants, their data, or biological material

Policy information about studies with human participants or human data. See also policy information about sex, gender (identity/presentation), and sexual orientation and race, ethnicity and racism.

Reporting on sex and gender	N/A
Reporting on race, ethnicity, or other socially relevant groupings	N/A
Population characteristics	N/A
Recruitment	N/A
Ethics oversight	N/A

Note that full information on the approval of the study protocol must also be provided in the manuscript.

Field-specific reporting

Please select the one below that is the best fit for your research. If you are not sure, read the appropriate sections before making your selection.

☐ Life sciences ☐ Behavioural & social sciences ☒ Ecological, evolutionary & environmental sciences

For a reference copy of the document with all sections, see nature.com/documents/nr-reporting-summary-flat.pdf

Ecological, evolutionary & environmental sciences study design

All studies must disclose on these points even when the disclosure is negative.

Study description	Analysis of microbial community composition, capabilities, and activities across two glacial foreland chronosequences in an alpine glacier, Switzerland and a maritime glacier, Antarctica
Research sample	Soil samples were collected from the forelands of two retreating glaciers: Sally Rocks tongue of Hurd Glacier on Livingston Island in Antarctica (March 2022) and Griessfirn Glacier in Canton Uri, Switzerland (August 2022).
Sampling strategy	For the Antarctic site, nine surface soil samples (0-10 cm) were collected across three soil age classes. Samples for 16S rRNA gene and metagenomic analyses were collected with a sterile spade, placed in whirl-pak sampling bags, frozen, and shipped to Monash University for processing. For Swiss samples, soils were collected at four soil age classes. In addition, at one location in each of the four soil-age classes, a 50-cm soil-profile was excavated to allow depth-resolved soil sampling at 10 cm intervals. Sampling locations were positioned along SW-NE-oriented transects that followed a single geomorphological landform, a band of lateral debris deposits, to minimize variability due to allogenic factors and intrinsic differences in microclimatic conditions and physical parameters among landform types. All samples were kept on ice or refrigerated until arrival in the laboratory and were thereafter stored at -20 °C (for molecular analysis) or 4 °C (for ex situ incubations) until further processing.
Data collection	Data collected included soil physicochemical analyses, in situ H2 / CO / CH4 fluxes, ex situ H2 / CO / CH4 / NH4 / S2- oxidation assays, community abundance by 16S rRNA gene qPCR, community composition by 16S rRNA gene amplicon sequencing, and community capabilities by genome-resolved metagenomics. The data collections are fully described in the methods.
Timing and spatial scale	Sampling relied on space-to-time substitution. The Antarctic samples corresponded to three soil age classes determined based on glacier front position measurements: A (< 5 yrs), B (10 yrs), and C (21 yrs). For the Swiss glacier, sampling locations were distributed along a well-defined soil chronosequence with increasing distance from the glacier terminus and categorized into four soil age classes: A (0-7yr), B (8-27yr), C (28-57yr) and D (58-127yr).
Data exclusions	No data or samples were excluded
Reproducibility	At each glacier, three surface soil samples (Antarctica), four surface soil samples (Switzerland; except site C where only three soils were collected) and depth transects (Switzerland; one per site) were collected. These biological replicates were analysed individually through soil physicochemical measurements, 16S rRNA gene amplicon sequencing, metagenomic sequencing, and ex situ biogeochemical assays. The sample size for all assays and analyses are provided in the manuscript. All attempts at reproduction were successful.
Randomization	No randomisation was conducted
Blinding	No blinding was conducted

Did the study involve field work? ☒ Yes ☐ No

Field work, collection and transport

Field conditions	All soils were collected during the summer under dry-weather conditions and distant from previous rainfall. The physicochemical characteristics of the soils collected are provided in Table S1.
Location	The Hurd Glacier is located at 62.6996°S, 60.4156°W, while the Griessfirn Glacier is at 46.84299°N, 8.82746°E. Specific coordinates for each sampling site at both glaciers are provided in Table S1.
Access & import/export	The Griessfirn Glacier samples were collected as part of a long-term study led by the Schroth Laboratory. The Antarctic sample collection was conducted under permit CPE-2020-9, issued by the Spanish Antarctic Committee, in accordance with Annex II of the Protocol on Environmental Protection to the Antarctic Treaty.
Disturbance	Sampling was conducted with utmost care to ensure minimal disturbance to the landscape, fauna, or flora. A low amount of sample was collected from each location (~200 grams). The depth profiles for the Swiss samples were filled in after sampling.

Reporting for specific materials, systems and methods

We require information from authors about some types of materials, experimental systems and methods used in many studies. Here, indicate whether each material, system or method listed is relevant to your study. If you are not sure if a list item applies to your research, read the appropriate section before selecting a response.

Materials & experimental systems

n/a	Involved in the study	
☒	☐	Antibodies
☒	☐	Eukaryotic cell lines
☒	☐	Palaeontology and archaeology
☒	☐	Animals and other organisms
☒	☐	Clinical data
☒	☐	Dual use research of concern
☒	☐	Plants

Methods

n/a	Involved in the study	
☒	☐	ChIP-seq
☒	☐	Flow cytometry
☒	☐	MRI-based neuroimaging

Plants

Seed stocks	N/A
Novel plant genotypes	N/A
Authentication	N/A
