# Supplementary material for: Metabolically flexible microorganisms rapidly establish glacial foreland ecosystems
Source: Nat Commun. 2025 Nov 26;16:11634. doi: 10.1038/s41467-025-66734-4 (PMC12749492; doi:10.1038/s41467-025-66734-4)
Supplement: Supplementary file 8 — Description Of Additional Supplementary File [file 41467_2025_66734_MOESM8_ESM.pdf]

Description of Additional supplementary files

Supplementary Data 1:

Soil physicochemistry and sample metadata. (a) Information about soil sample location, depth, age, altitude, and coordinates. (b) Environmental parameters for each Antarctic and Swiss soil sample.

Supplementary Data 2:

16S community analysis & chlorophyll a measurement. (a) qPCR results (b-c) 16S rRNA gene ASVs table and statistics. (d) Community richness based on 16S rRNA gene. (e) Test for significant differences in community structure based on 16S rRNA gene. (f) Chlorophyll a data. (g) Specialization index genus level for the Antarctic foreland samples. (h) Specialization index genus level for the Swiss foreland samples.

Supplementary Data 3 :

Metagenomic analysis. (a) Short-read data showing 56 metabolic marker genes in the Antarctic soil samples. (b) Short-read data showing 56 metabolic marker genes in the Swiss soil samples. (c) Generalised-linear model analysis showing difference in selected metabolic marker genes abundance across the two glacier forelands. (d) Random forest analysis of environmental drivers of selected marker genes. (e) Metagenome-assembled genome data of the Antarctic glacier including taxonomic classification, quality, coverage, niche breadth, and metabolic markers presence. (f) Metagenome-assembled genome data of the Swiss glacier including taxonomic classification, Done quality, coverage, niche breadth, and metabolic markers presence. (g) Statistic showing discrimination among habitat generalist, specialist, and intermediate in the Antarctic and Swiss forelands.(h) Gene descriptions and diamond search parameters.

Supplementary Data 4 :

Description: MAG species index and metabolic modules. (a) Niche breadth of Antarctic metagenome-assembled genomes. (b) Niche breadth of Swiss metagenome-assembled genomes. (c) Abundance of habitat generalist, specialist, and intermediate across the Antarctic chronosequence. (d) Abundance of metabolic modules in Antarctic metagenome-assembled genomes. (e) Abundance 56 of metabolic marker gene in each Antarctic metagenome-assembled genomes. (f) Abundance of habitat generalist, specialist, and intermediate across the Swiss chronosequence. (g) Abundance of metabolic modules in Swiss metagenome-assembled genomes. (h) Abundance 56 of metabolic marker gene in each Swiss metagenome-assembled genomes. (i) Abundance of metabolic modules across Antarctic and Swiss habitat generalist, specialist, and intermediate corrected metagenome-assembled genomes count and by average completeness.

Supplementary Data 5 :

Gas oxidation rates, in situ fluxes and oxic slurry nutrient assays. (a) Ex situ trace gas oxidation rates and cell specific power calculations. (b) In situ trace gas soil to atmosphere fluxes. (c) uptake and accumulation rates of nitrogen and sulfur species.
